# Vogt-Koyanagi-Harada Syndrome: A Case Report

**DOI:** 10.7759/cureus.64702

**Published:** 2024-07-16

**Authors:** Thummalagunta Prathyusha, Mohammad Asif, Sai T Gadde

**Affiliations:** 1 General Medicine, All India Institute of Medical Sciences, Mangalagiri, Guntur, IND

**Keywords:** immunosuppressive therapy, autoimmune, neurological disorders, rare disease, vogt-koyanagi-harada disease

## Abstract

Vogt-Koyanagi-Harada disease (VKH) is a neurological disorder that impacts vision and hearing by causing the immune system to attack melanocytes. Symptoms of the condition include flu-like symptoms, eye pain, headache, and dizziness, which may progress to vitiligo and hearing impairment. The diagnostic criteria include ocular involvement, generalized choroiditis, tinnitus, meningitis, and skin depigmentation. The treatment includes corticosteroids and immunosuppressive drugs. VKH is believed to be an autoimmune condition, possibly triggered by hereditary factors and cross-reactivity with cytomegalovirus. VKH is common in East Asia and India and has a genetic link to certain alleles. Inflammation generated by Th1 in melanocytes results in the production of granulomas. An analysis of a 48-year-old female with VKH disease revealed symptoms of anterior uveitis and subsequent glaucoma. The treatment involved the administration of systemic steroids and intratympanic steroid injections. Biochemical indicators showed signs of inflammation. Timely identification and therapy are essential for managing VKH. Further research is necessary to enhance outcomes for patients with VKH disease.

## Introduction

Vogt-Koyanagi-Harada (VKH) disease is a neurological condition that affects vision and hearing. It is an infrequent autoimmune inflammatory condition that predominantly impacts tissues containing melanocytes, including the eyes, skin, inner ear, and meninges.

Alfred Vogt first described it in 1906, followed by Yoshizo Koyanagi in 1926 and Einosuke Harada in 1908. The disease causes a loss of immune tolerance to melanocytes in various body parts. Despite its unique phenotype, the pathophysiologic mechanisms remain challenging. Early identification and treatment can reduce morbidity, and the prognosis ranges from minimal to severe vision loss [1]. The condition presents with symptoms such as the flu, including eye discomfort, headache, and dizziness, and may lead to vitiligo and hearing impairment. Diagnostic criteria include no history of ocular trauma or surgery, bilateral ocular involvement, diffuse choroiditis, tinnitus, meningismus, and skin depigmentation. Although the precise etiology of VKH disease remains incompletely elucidated, it is hypothesized to entail an aberrant immune reaction that specifically targets melanocytes in patients with a hereditary predisposition. The etiology of VKH encompasses various factors, such as an autoimmune reaction, hereditary susceptibility, molecular mimicry, and immune-mediated inflammation [2].

Inflammation of the uvea within the ocular region can cause many symptoms, including photophobia, blurred vision, and ocular pain. Similarly, in the integumentary system, depigmentation and the formation of skin lesions may manifest. VKH disease is distinguished by its systemic effects that extend beyond the ocular region, presenting with neurological symptoms, hearing impairments, and dermatological indications. Meningeal inflammation can result in aseptic meningitis, a condition that can cause intense headaches and neurological impairments [3].

VKH disease is a multifaceted ailment characterized by dysregulated immune responses that specifically target melanocytes in diverse tissues. This results in inflammation and subsequent tissue impairment. The standard approach to management usually entails using immunosuppressive treatment to regulate inflammation and mitigate the risk of enduring problems [4].

Treatment involves corticosteroid therapy and biological and immunosuppressive medications to suppress disease activity and improve symptoms [5]. VKH is hypothesized to be caused by an autoimmune response to melanocyte-associated antigens in genetically predisposed individuals. Patients with VKH disease-associated T cells show cross-reactivity with cytomegalovirus (CMV)-specific sequences and tyrosinase protein and exhibit autoimmunity targeting retinal components. Diagnosis depends on clinical characteristics, and therapy involves high-dose systemic corticosteroids over six months [6].

VKH is a prevalent cause of uveitis throughout both Thailand and India, with a prevalence of 21.08% in India. Moreover, approximately 800 new cases are reported in Japan annually. VKH is uncommon in the United States, representing 3-4% of admissions to specialized medical centers. North American cases reveal a prevalence of Hispanic and Native American populations, but South American nations such as Colombia have not experienced a notable rise in VKH incidence. Genetic research indicates that the DRB1-04*01 allele is more common in East Asian people and that HLA-DRB4 or HLA-DRB1 alleles are more closely associated with VKH disease. Patients with VKH disease are usually in their third or subsequent decade of life, with cases in children being generally severe and difficult to manage [7].

Th1-induced inflammation occurs in melanocytes, particularly in HLA DRB1-04*05 melanocytes, due to environmental stimuli. The tyrosinase peptide, which is targeted by T cells, is similar to CMV peptides. The inflammatory process results in the formation of non-necrotizing granulomas, such as Dalen-Fuchs nodules in the eye’s lens, which can also occur in sympathetic ophthalmia [7].

## Case presentation

A 48-year-old female day-wage laborer presented to the emergency department at All India Institute of Medical Sciences, Mangalagiri with complaints of headaches and bilateral eye pain for the past 15 days. She had visited the hospital multiple times, seeking relief for her visual disturbances and ocular congestion, but without success. She was diagnosed with anterior uveitis, progressive glaucoma, and VKH disease following a comprehensive examination and diagnostic workup that provided us with the necessary information.

The patient experienced headaches associated with photophobia, hearing loss, and tinnitus. She also reported a recent episode of mild fever and generalized body itching. Particularly noteworthy was the fact that she had a history of COVID-19 infection, as well as two previous cesarean procedures. She had reached menopause two years earlier. During the examination, her vital signs were within normal ranges, but neurological signs indicated potential brain involvement, warranting further investigation.

From day 1 to day 15, the patient experienced headaches and bilateral eye pain. On day 16, she underwent a full physical examination, which included an MRI of the brain. The scan showed problems with the pituitary gland and higher levels of inflammatory markers, specifically C-reactive protein (CRP) and total leukocyte count (TLC). On day 17, following the MRI findings, a cerebrospinal fluid (CSF) analysis was performed, revealing pleocytosis consistent with VKH. Additional imaging supported the diagnosis, which was confirmed on day 18 based on clinical features and diagnostic criteria. On day 18, the diagnosis of VKH was confirmed based on clinical features and diagnostic criteria.

The diagnosis of VKH was confirmed by the following criteria: no history of recent bilateral ocular surgery or trauma, presence of diffuse choroiditis observed through imaging studies, tinnitus and other auditory symptoms such as hearing loss, meningismus indicating meningeal involvement, and dermatological signs including skin depigmentation. High levels of TLC, CRP, total bilirubin, and serum globulin were found in the lab tests. The patient tested positive for hepatitis C virus, while tests for human immunodeficiency virus and tuberculosis were negative.

Table 1 shows the laboratory data of the patient presenting with symptoms suggestive of VKH disease, along with anterior uveitis and secondary glaucoma. Laboratory investigations and imaging ruled out tuberculosis, as features seen in VKH can overlap with other uveo-meningeal syndromes. Parameters include various hematological, biochemical, and immunological markers obtained during investigations.

**Table 1:** Laboratory data for the patient presenting with Vogt-Koyanagi-Harada disease and anterior uveitis.

Parameter	Result	Normal range
Hemoglobin	11.4 g/dL	12.0–15.5 g/dL
Total leukocyte count	21.5 × 10^3^/µL	4.0–11.0 × 10^3^/µL
Platelet Count	400 × 10^3^/µL	150–450 × 10^3^/µL
C-reactive protein	75.6 mg/L	< 10 mg/L
Erythrocyte sedimentation rate	25 mm/hour	0–20 mm/hour
Procalcitonin	0.07 ng/mL	<0.5 ng/mL
Total bilirubin	3.4 mg/dL	0.2–1.0 mg/dL
Serum albumin	3.1 g/dL	3.5–5.5 g/dL
Serum globulin	4.5 g/dL	2.0–3.5 g/dL
Blood urea	59 mg/dL	10–50 mg/dL
Serum potassium	5.8 mmol/L	3.5–5.1 mmol/L
Angiotensin-converting enzyme	13.5 U/L	8.3–21.4 U/L
Urine culture and sensitivity	Within normal limits	-
Thyroid-stimulating hormone	1.8 µIU/mL	0.4–4.0 µIU/mL
Hemoglobin A1c	3.9%	4.0–5.6%
Absolute CD3	1,081 cells/µL	-
Absolute CD4	698 cells/µL	-
Absolute CD8	317 cells/µL	-
CD4/CD8 ratio	2.20	-
Toxoplasma IgM	Negative	-
Treponema pallidum hemagglutination assay	Negative	-
Scrub typhus IgG	Negative	-
Scrub typhus IgM	Negative	-

**Figure 1:**
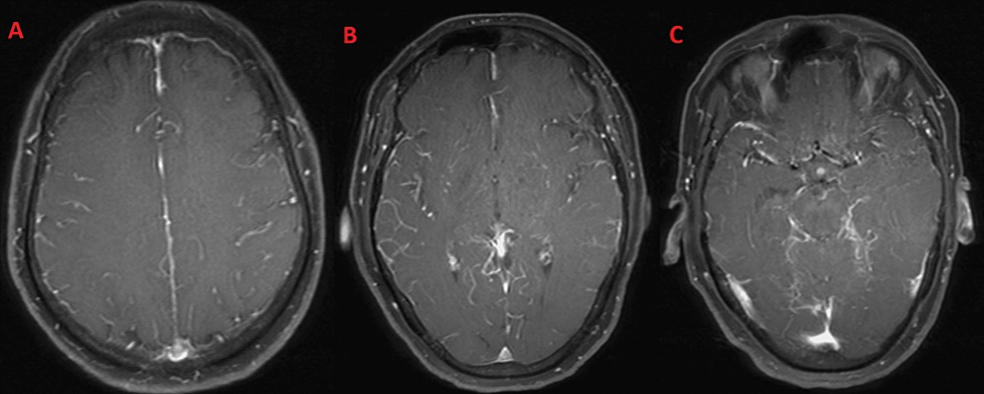
A-C: Axial T1 fat-saturated post-contrast images demonstrating diffuse leptomeningeal enhancement along the sulcal spaces and cisterns.

**Figure 2:**
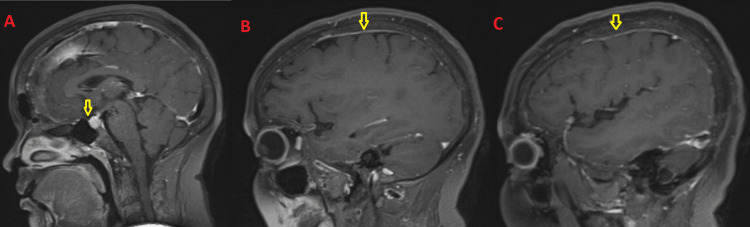
MRI scan of the brain. A: The pituitary is mildly bulky (10 mm) with a mildly thickened enhancing stalk (arrows). B, C: There is smooth enhancing pachymeningeal thickening along the bilateral cerebral convexities (arrows).

Leptomeningeal augmentation and pituitary abnormalities were among the positive results on the brain MRI discovered during the investigation. Additionally, higher inflammatory markers were found. The neurology department suggested conducting additional tests, such as analyzing the CSF and imaging scans, which ultimately led to the beginning of the treatment with systemic steroids (Figures 1, 2).

The patient was treated symptomatically with scabies treatment and antibiotics (doxycycline, ceftriaxone, Magnex Forte, acyclovir) to alleviate the symptoms of broad itching, and she was advised to follow up after two weeks. A dermatological consultation was also provided to address widespread irritation and recommendations were given.

The patient received two doses of intratympanic injection of methylprednisolone, with the next treatment scheduled for a future date. The patient’s symptoms improved, and she was discharged in a hemodynamically stable condition. Furthermore, an ENT consultation was sought to manage hearing loss, which included the administration of intratympanic steroid injections. Although she was making progress, she continued to have some of the same symptoms.

The white blood cell count of the patient remained elevated during her entire hospital stay, and it did not begin to decrease until after both the third and fourth doses of intratympanic steroids were administered. In the end, her symptoms improved and she was released from the hospital in a stable condition.

This case depicts the difficulties of diagnosing and treating complicated autoimmune illnesses such as VKH. To give complete care to the patient, a multidisciplinary strategy that involved a variety of medical disciplines was essential. This highlights the significance of teamwork in treating such complicated medical diseases.

## Discussion

The symptoms of VKH presented by our patient show similarity to the findings of a study by Kaza et al. (2021) [8], providing insights into the clinical symptoms and consequences of VKH syndrome in pediatric patients. Kaza et al. discovered that a sizeable number of patients continued to experience symptoms, with the average duration of these symptoms exceeding 10 weeks. The difficulties associated with the management of VKH syndrome are highlighted by these findings, particularly in younger demographics.

There are distinct phases associated with VKH disease. These phases include prodromal, uveitic, convalescent, and recurring stages, presenting clinical characteristics and difficulties. Extraocular signs such as migraines, meningismus, and hearing loss further complicate the clinical picture, highlighting the fact that the disease is systemic in origin [9]. O’Keefe and Rao (2017) [9] observed that the diagnosis and treatment of VKH disease have been completely transformed by advancements in imaging technologies. Clinicians can visualize retinal detachments and choroidal alterations that are diagnostic of VKH syndrome using ocular coherence tomography, which has emerged as a significant technique for early detection and monitoring.

Corticosteroids are often used to treat VKH disease and for acute control. This is then followed by immunomodulatory therapy for a longer period to prevent recurrence and consequences. Prompt and aggressive management is crucial to obtain favorable outcomes and reduce the risk of problems such as cataracts, glaucoma, and sunset glow fundus.

The proper management of VKH syndrome continues to be difficult despite the breakthroughs made in therapeutics. The fact that clinical manifestations and responses to treatment might vary greatly from patient to patient highlights the importance of providing individualized care customized to each patient’s specific symptoms and clinical progression. Our case report is consistent with the findings of the studies by Kaza et al. (2021) [8] and O’Keefe and Rao (2017) [9] and offers useful insights into the medical characteristics, diagnostic methods, and therapeutic strategies for VKH syndrome. There is a need for additional research to be conducted to enhance treatment regimens and improve results, particularly in pediatric populations, where the condition can have substantial long-term repercussions.

The research on the placement of cochlear implants in VKH syndrome patients provided information on the difficulties and outcomes linked with this complicated inflammatory disorder. In the setting of cochlear implantation, the VKH condition, which is characterized by inflammation in several systems, including panuveitis and neurological symptoms, presents a set of specific concerns to consider. A case report that detailed the experience of a 30-year-old Saudi woman shed light on the potential problems that may arise during cochlear implantation in patients with VKH syndrome as well as the eventual success that can be achieved [10].

After more than five years of follow-up, the patient’s cochlear implantation treatment resulted in an outstanding hearing restoration. This was the case despite the patient’s ongoing visual complaints and the advancement of the ocular condition. On the other hand, the technique was not difficult, as the sequential cochlear implantation was made more difficult by intracochlear degeneration. This finding highlights the importance of carefully diagnosing and managing VKH patients undergoing cochlear implantation. This is especially important because of the autoimmune nature of the disorder, which may make individuals more susceptible to fibrotic alterations in the cochlea [10].

Our case of a 48-year-old woman who presented with symptoms that were suggestive of VKH syndrome further emphasizes the multisystemic character of this condition and the significance of comprehensive examination and care. Her symptoms, which included headaches, photophobia, hearing loss, and itching, highlight the numerous clinical manifestations of VKH syndrome. As a result, a multidisciplinary approach is required to diagnose and treat the condition.

As a result of the diagnostic workup, leptomeningeal enhancement and pituitary abnormalities were discovered on the MRI. Additionally, higher inflammatory markers were found, which indicated that VKH syndrome was involved throughout the body. Owing to treatment with systemic steroids, in addition to scabies therapy and antibiotics for symptomatic relief, the patient’s symptoms improved, and she was discharged in a stable state. Nevertheless, the fact that certain symptoms continue to be present despite treatment highlights the difficulty of managing VKH syndrome as well as the indispensable requirement for continuous monitoring and intervention.

Furthermore, the link between VKH syndrome and primary open-angle glaucoma in a 62-year-old female patient with diabetes mellitus brings to light the difficulties associated with the management of comorbid illnesses as well as the potential limitations of corticosteroid treatment in diabetic patients. The necessity of individualized treatment approaches and thorough monitoring in patients with VKH who have complex medical histories is highlighted by this particular case [11].

Our case report highlights the importance of fully understanding VKH syndrome and the potential issues it may cause in clinical settings. When optimizing results and enhancing the quality of life for individuals with VKH syndrome, engaging in multidisciplinary teamwork, conducting meticulous evaluations, and developing individualized treatment plans are all crucial components. It is necessary to conduct additional research to better understand the underlying mechanisms and the most effective management strategies for this complicated autoimmune condition.

## Conclusions

VKH disease is a rare autoimmune condition affecting the visual, auditory, and cutaneous systems due to immunological attacks on melanocytes. Common in East Asia and India, VKH symptoms include eye pain, headache, and dizziness, progressing to vitiligo and hearing loss. Genetic factors and cross-reactivity with CMV are potential triggers. Diagnostic criteria include choroiditis and skin depigmentation, and treatment primarily involves immunosuppression. Our case of a 48-year-old woman with VKH highlights the complexity of the condition and the importance of a multidisciplinary approach to diagnosis and treatment. Managing comorbidities such as primary open-angle glaucoma is crucial, emphasizing tailored treatment strategies and vigilant monitoring. Further research is needed to improve outcomes for VKH patients.
